# Polarity Directed Appraisal of Pharmacological Potential and HPLC-DAD Based Phytochemical Profiling of *Polygonum glabrum* Willd

**DOI:** 10.3390/molecules27020474

**Published:** 2022-01-12

**Authors:** Tahira Sultana, Madiha Ahmed, Nosheen Akhtar, Mohammad K. Okla, Abdulrahman Al-Hashimi, Wahidah H. Al-Qahtani, Hamada Abdelgawad, Ihsan-ul-Haq

**Affiliations:** 1 Department of Pharmacy, Faculty of Biological Sciences, Quaid-i-Azam University, Islamabad 45320, Pakistan; tahira.sultana69@gmail.com; 2 Shifa College of Pharmaceutical Sciences, Shifa Tameer-e-Millat University, Islamabad 44000, Pakistan; 3 Department of Biological Sciences, National University of Medical Sciences, Rawalpindi 43600, Pakistan; nosheenakhtar@numspak.edu.pk; 4 Botany and Microbiology Department, College of Science, King Saud University, Riyadh 11451, Saudi Arabia; okla103@yahoo.com (M.K.O.); aalhashimi@ksu.edu.sa (A.A.-H.); 5 Department of Food Sciences and Nutrition, College of Food and Agriculture Sciences, King Saud University, Riyadh 11451, Saudi Arabia; wahida@ksu.edu.sa; 6 Integrated Molecular Plant Physiology Research, Department of Biology, University of Antwerp, 2020 Antwerpen, Belgium; hamada.abdelgawad@uantwerpen.be

**Keywords:** *Polygonum glabrum*, protein kinase inhibition, α-amylase inhibition, antimicrobial plants

## Abstract

The present study was designed to evaluate polarity-dependent extraction efficiency and pharmacological profiling of *Polygonum glabrum* Willd. Crude extracts of leaves, roots, stems, and seeds, prepared from solvents of varying polarities, were subjected to phytochemical, antioxidant, antibacterial, antifungal, antidiabetic, and cytotoxicity assays. Maximum extraction yield (20.0% *w*/*w*) was observed in the case of an acetone:methanol (AC:M) root extract. Distilled water:methanol (W:M) leaves extract showed maximum phenolic contents. Maximum flavonoid content and free radical scavenging potential were found in methanolic (M) seed extract. HPLC-DAD quantification displayed the manifestation of substantial quantities of quercetin, rutin, gallic acid, quercetin, catechin, and kaempferol in various extracts. The highest ascorbic acid equivalent total antioxidant capacity and reducing power potential was found in distilled water roots and W:M leaf extracts, respectively. Chloroform (C) seeds extract produced a maximum zone of inhibition against *Salmonella typhimurium*. Promising protein kinase inhibition and antifungal activity against *Mucor* sp. were demonstrated by C leaf extract. AC:M leaves extract exhibited significant cytotoxic capability against brine shrimp larvae and α-amylase inhibition. Present results suggest that the nature of pharmacological responses depends upon the polarity of extraction solvents and parts of the plant used. *P. glabrum* can be considered as a potential candidate for the isolation of bioactive compounds with profound therapeutic importance.

## 1. Introduction

Plant extracts are being used to cure various ailments since antiquity. According to estimates, more than 70% of people in the world still rely on traditional medicinal plants for their primary health care needs. Plants being an unremitting source of lead compounds have been exploited for the identification and isolation of significant secondary metabolites which have been included in modern therapeutic regimens, including quinine, artemisinin, vincristine, vinblastine, paclitaxel, etc. [1]. The herbal extracts that are usually originated and used by the indigenous communities have continuously been monitored over generations by their efficacy and side effects [2]. However, systematic scientific data on the efficacy, mode of action, and active constituents are still under development. Recent times have seen a growing interest in such investigations of the plant kingdom to ascertain the activity of the extracts and categorize active compounds [3].

*Polygonum glabrum* belongs to the family Polygonaceae. This family consists of nearly 50 genera with 1120 species, including herbs (both monoecious and dioecious), shrubs, and small trees [4]. The genus *Polygonum* L. has almost 300 species around the world in temperate climates [5]. This genus is well known for producing a wide variety of bioactive compounds, e.g., tannins, triterpenoids, anthraquinones, coumarins, phenylpropanoids, lignans, and flavonoids, as the dominating group of constituents [5,6,7,8,9]. *P. glabrum* is mostly found on the river banks, marshy areas, and streamside in the form of clumps. In traditional practice, its roots are used in piles, debility, consumption, and jaundice. *P. glabrum* is reported to have good activity against *Micrococcus pyrogens* and *Diplococcus pyrogens* [8,10]. The screening of the chloroform extract indicated the presence of some alkaloids, carbohydrates, and flavonoids [11]. However, a comprehensive review of the literature suggests that a colossal effort to ascertain the curative perspective of individual parts of *P. glabrum* by using an extensive array of bioassays is yet to be placed.

The current study has exploited a wide variety of extraction solvents having varying polarities to observe their effects on extraction productivity and bioactivity. Furthermore, our interest was to explore the correlation between different phytochemicals and antioxidant, antimicrobial, cytotoxic, as well as antidiabetic behavior of stem, seeds, leaves, and roots of *P. glabrum*. To the best of our knowledge, polarity-dependent extensive study along with protein kinase and α-amylase inhibition potential has been done and reported for the first time on this plant.

## 2. Material and Method

### 2.1. Preparation of Crude Extracts

After collection of the plant from Quaid-i-Azam University, Islamabad, Pakistan, in October 2019, identification was carried out, and the plant specimen was documented by the herbarium number PHM-488 in the herbarium of medicinal plants, Quaid-i-Azam University, Islamabad, Pakistan.

For the preparation of test extracts, different plant parts (leaves, stems, roots, and seeds) were separately washed under running tap water to eliminate waste products such as dust, etc., shade dried for 6 weeks and ground afterward. The finely powdered material of all parts was soaked using the maceration process with continuous sonication separately in 14 solvent systems, including *n*-hexane (NH), chloroform (CH), Ethyl acetate (EA), Acetone (AC), Methanol (M), Ethanol (ET), Distilled water (W), *n*-hexane:Ethyl acetate (NH:EA), *n*-hexane:Ethanol (NH:ET), Chloroform:Methanol (CH:M), Ethyl acetate:Methanol (EA:M), Acetone:Methanol (AC:M), Distilled water:acetone (W:AC), and Distilled water:Methanol (W:M). Sixty grams of powdered material was macerated in 250 mL of each solvent system followed by ultrasonication 3–5 times a day at room temperature for 5 min. This procedure was repeated three times by combining all the resulting filtrates, followed by drying using a rotary evaporator. It was then placed under a vacuum at 45 °C for the removal of residual traces of solvents. The crude extracts, ready for final use, were then stored at −20 °C till further usage. Dried crude extracts were weighed using weighing balance, and percent extract recovery was calculated using the following formula:

$$\mathrm{Percent}\;\mathrm{extract}\;\mathrm{recovery}=\frac{\mathrm{We}}{\mathrm{Wp}}\times 100$$

where Wp = Weight of dried powder and We = Weight of the crude extract.

### 2.2. Phytochemical Analysis

#### 2.2.1. Total Phenolic Content (TPC)

Previously described protocol was followed to assess total phenolic content [12]. Gallic acid was used as a positive control, while DMSO was used as a negative control. For each run, the reaction mixture comprising an aliquot of 20 μL test sample (4 mg/mL in DMSO), 90 μL of Folin-Ciocalteu reagent (10 times diluted), and 90 μL of Na_2_CO_3_ (6% *w*/*v*) was incubated for 30 min, and absorbance was taken at 630 nm. Different concentrations of Gallic acid (50, 25, 12.5, and 6.25 µg/mL) were used to develop calibration curve (y = 0.639x + 0.556; R^2^ = 0.9717) to calculate µg Gallic acid equivalents (GAE). The assay was run three times under the same experimental conditions, and the results were expressed as average µg GAE/mg extract.

#### 2.2.2. Total Flavonoid Content (TFC)

To determine the total flavonoid contents, a previously described protocol was followed [12]. The reaction mixture contained an aliquot of 20 μL of the test sample (4 mg/mL), 10 μL of 1 M CH_3_COOK, 10 μL of AlCl_3_ solution (10% *w*/*v*), and 160 μL of distilled water. It was incubated for 30 min at room temperature, after which the absorption was measured at 415 nm. A calibration curve (y = 0.117x + 0.0687, R^2^ = 0.993) was developed using varying concentrations (6.25–50 μg/mL) of quercetin to calculate quercetin equivalents (QE) in the reaction mixture. The assay was run three times under the same experimental conditions, and the flavonoid contents were expressed as average μg QE/mg extract.

#### 2.2.3. Quantitative Analysis by HPLC-DAD

The previously reported protocol was followed for the HPLC-DAD analysis [13]. HPLC (Agilent 1200 series with binary gradient pump) equipped with C8 analytical column and DAD (Agilent technologies, 156 Germany) was used. Stock solutions of 8 standards were prepared with methanol to obtain the final concentration of 50 µg/mL each. Methanol was used for the preparation of test samples (10 mg/mL). The test samples were first dissolved in methanol, followed by sonication for 5 min. Finally, the test solutions were filtered through 0.2 μm sartolon polyamide membrane filter to remove any impurity left. The solutions were freshly set just before analysis and were kept at 4 °C till further analysis. Two mobile phases were used for the identification and quantification of polyphenols, i.e., mobile phase ‘A’ contained Acetonitrile:methanol:water:acetic acid in a ratio of 5:10:85:1 while mobile phase ‘B’ contained acetonitrile:methanol:acetic acid in a ratio of 40:60:1. The flow rate was kept at 1 mL/min. Aliquots of 20 μL of each sample solution were injected through the injector into the column. The column was reconditioned 10 min before running each sample. The gradient volume of mobile phase B was 0–50% in 0–20 min, 50–100% in 20–25 min finally 100% from 25 to 30 min. The standard compounds were employed in the preparation of stock solutions and subsequent dilutions of varying concentrations, i.e., 12.5, 25, 50, and 100 μg/mL. The calibration curves of each standard were prepared using the data of peak area and concentrations. Subsequently, the same data was utilized to carry out the regression analysis and for the calculations of the limit of detection (LOD) and limit of quantification (LOQ) values. The absorbance of test samples was taken at respective wavelengths, i.e., 257 nm for rutin and gallic acid, 279 nm for catechin, 325 nm for caffeic acid and apigenin, 368 nm for kaempferol, myricetin, and quercetin.

### 2.3. Pharmacological Evaluation

#### 2.3.1. Antioxidant Assays

##### DPPH Radical Scavenging Assay

Free radical scavenging activity was performed by following the previously described procedure [12]. Briefly, 10 μL of each test solution with different dilutions was mixed with 190 μL of DPPH solution (9.2 mg/100 mL in methanol) in each well of 96 well plates so that to have final concentrations of 200, 66.66, 22.22, and 7.41 µg/mL. The reaction mixture was incubated at 37 °C for 1 h in a dark area, and the absorbance was taken at 515 nm. The same protocol was followed for the positive control (ascorbic acid at final concentrations of 20, 6.66, 2.22, and 0.74 μg/mL) and negative control (DMSO). Percent DPPH free radical scavenging activity (%FRSA) was determined by the following formula:

$$\%\mathrm{FRSA}=\left(1-\frac{\mathrm{As}}{\mathrm{Ac}}\right)\times 100$$

where As is the absorbance of the sample while Ac is the absorbance of the negative control. The assay was run three times. IC_50_ values were also calculated for the test samples, which reported radical scavenging activity of greater than 50%.

##### Total Reducing Power (TRP) Determination

The reducing potential of test samples of *P. glabrum* was assessed by a previously described protocol [12]. The reaction mixture containing aliquots of 200 μL of samples (4 mg/mL DMSO), 400 μL of each phosphate buffer (0.2 mol/L, pH 6.6) and potassium ferricyanide (1% *w*/*v*) was incubated at 50 °C for 20–30 min followed by the addition of 400 µL of trichloroacetic acid (10% *w*/*v*). It was then centrifuged for 10 min at 3000 rpm at room temperature. Distilled water (500 µL) and FeCl_3_ solution (100 µL, 0.1% *w*/*v*) was added to the upper layer of centrifuged solution. The absorbance of the plate was taken at 630 nm. A calibration curve (y = 0.388x + 0.19, R^2^ = 0.9868) of ascorbic acid was obtained at different concentrations (100, 50, 25, 12.5, and 6.25 µg/mL) and the resultant reducing power of each sample was expressed as µg AAE/mg extract. The experiment was done three times under the same experimental conditions.

##### Total Antioxidant Capacity (TAC) Determination

To evaluate the samples for total antioxidant capacity, a previously described phosphomolybdenum based protocol was followed [12]. The reaction mixture was set by adding aliquots of 100 μL of test extract (4 mg/mL), 900 μL of reagent solution containing 28 mM NaH_2_PO_4_, 4 mM ammonium molybdate, and 0.6 M H_2_SO_4_. The reaction mixture was incubated at 95 °C for 90 min followed by cooling at ambient temperature. The absorbance of the reaction mixture was taken at 630 nm. Ascorbic acid was employed as a positive control, while DMSO was used as a negative control. Different final concentrations of ascorbic acid (100, 50, 25, and 12.5 μg/mL) were utilized to develop a standard curve (y = 0.51x + 0.61; R^2^ = 0.995). The experiment was done three times. The final results were expressed as μg AAE/mg extract.

#### 2.3.2. Antimicrobial Assays

##### Antibacterial Assay

Standard agar disc diffusion method was followed for in vitro antibacterial potential evaluation [13] against four bacterial strains, including *Micrococcus luteus*, *Staphylococcus aureus, Salmonella typhimurium*, and *Klebsiella pneumoniae*. Turbidity of 24 h refreshed microbial culture was standardized as per 0.5 McFarland turbidity standard. To grow bacterial lawn on the sterile nutrient agar plates, the refreshed bacterial culture (50 μL) of 1 × 10^6^ CFU/mL seeding density was poured onto the solidified agar plates and swabbed by using a sterile cotton bud. Sterile filter paper discs (6 mm diameter) permeated with sample (5 μL of 20 mg/mL), a positive control (5 μL of 4 mg/mL cefixime and ciprofloxacin), and a negative control (DMSO) were placed on agar plates followed by incubation for a time period of 24 h at 37 °C. After incubation, a zone of growth inhibition around each disc was observed and measured. Each sample was tested in triplicate.

Samples were also subjected to minimum inhibitory concentration (MIC) determination by the broth microdilution method [13]. Briefly, bacterial inoculum (190 µL) at a density of 5 × 10^2^ CFU/mL, in nutrient broth was incubated with samples (final concentrations of 200, 66.6, 22.2 and 7.4 µg/mL), at 37 °C for 30 min. Afterward, absorbance was measured at 600 nm at day 0, followed by the incubation and re-measurement of the absorbance after 24 h. Results were calculated using the following formula:

$$\%\mathrm{Inhibition}\;\mathrm{of}\;\mathrm{growth}=\left(1-\frac{\mathrm{Ts}}{\mathrm{Tc}}\right)\times 100$$

where Ts and Tc represent the turbidity of the sample and control, respectively.

##### Antifungal Assay

Antifungal potential of the test extracts was assessed using the standard agar disc diffusion method [13] against five fungal strains, i.e., *Fusarium solani*, *Aspergillus fumigatus*, *Aspergillus niger*, *Aspergillus flavus*, and *Mucor* sp. An aliquot of 100 μL of freshly prepared standardized spore suspension harvested in 0.02% *v*/*v* Tween 20 solution was poured and swabbed on the sterile Sabraud dextrose agar plates. Then 5 μL of sample was loaded on the sterile disc (6 mm) and mounted on the seeded plate. Similarly, clotrimazole (5 μL of 4 mg/mL) was used as positive control, while DMSO was used as a negative control. All the plates were kept for incubation at 28 °C for 24–48 h. The zone of inhibitions around each disc was observed and measured. Each sample was tested in triplicates. Samples having significant activity were further exploited in an assay for MIC determination.

#### 2.3.3. Enzyme Inhibition Assay

##### α-Amylase Inhibition Assay

α-amylase inhibition potential of test samples was evaluated by following the previously reported standard protocol [12] with minor modifications. Briefly, 15 µL phosphate buffer (pH 6.8), 25 µL alpha amylase enzyme (0.14 U/mL), 10 µL of the test sample (4 mg/mL in DMSO) and 40 µL starch (2 mg/mL in phosphate buffer) was added to the wells of 96-well plates to prepare a reaction mixture. After incubation at 50 °C for 30 min, an aliquot of 20 μL of 1 M HCl was added to halt the reaction, followed by the addition of 90 μL of iodine solution (5 mM iodine and 5 mM potassium iodide) to each well. A blank (containing buffer solution, starch, and DMSO), a negative and positive control containing DMSO and acarbose (250 µM), respectively, instead of sample, were included in the assay. Absorbance measurements were carried out at 540 nm, and the results were expressed as % α-amylase inhibition/mg extract. The following formula was used for the calculation of percent α-amylase inhibition:

$$\%\;\alpha -\mathrm{amylase}\;\mathrm{inhibition}=\left[\frac{\mathrm{OD}\;\mathrm{of}\;\mathrm{sample}-\mathrm{OD}\;\mathrm{of}\;\mathrm{negative}\;\mathrm{control}}{\mathrm{OD}\;\mathrm{of}\;\mathrm{blank}\;-\mathrm{OD}\;\mathrm{of}\;\mathrm{negative}\;\mathrm{control}}\right]\times 100$$

#### 2.3.4. Cytotoxicity Assays

##### Brine Shrimp Lethality Assay

A 24 h lethality test was performed against brine shrimp (*Artemia salina*) larvae in accordance with a previously described protocol [12]. Brine shrimps were hatched in simulated seawater (38 g/L supplemented with 6 mg/L dried yeast) by incubating *A. salina* eggs (Ocean 90, USA) for 24–48 h in a two-compartment plastic tray under light, providing direct illumination and heat (30–32 °C). Pasteur pipette was used for the harvesting of the mature nauplii, and 10 nauplii were transmitted to each well of the 96 well plate. Test samples were initially tested at two-fold concentrations, i.e., 500, 250, 125, 63, and 32 µg/mL, by transferring the equivalent volume of each test solution into the wells containing seawater and nauplii. Doxorubicin (at final concentrations 50, 25, 12.5, 6.25, and 3.125 µg/mL) was used as a positive control while DMSO was used as a negative control. It was kept for incubation for 24 h followed by the counting of the surviving nauplii. The percentage of deaths for each well was determined against each dose and control. The experiment was run three times.

##### Protein Kinase Inhibition (PKI) Assay

Assessment of test samples against *Streptomyces* 85E test strain was done by following the previously described protocol [14]. *Stremtomyces* 85E spores were refreshed using sterile tryptone soya broth (TSB) by incubating at 28 °C for 24 h. The refreshed culture (100 μL) was seeded on the sterile plates containing sterile ISP4 medium (prepared in the lab) and swabbed by using a sterile cotton bud. The sterile filter paper discs (6 mm) were permeated with 5 μL test solution (20 mg/mL in DMSO) and mounted on a freshly seeded media plate. Surfactin (5 μL from 4 mg/mL solution) loaded disc was maintained as positive control while DMSO loaded disc was used as a negative control. Petri plates were kept for incubation at 28 °C for 72–96 h to allow the hyphae development. After the incubation period, the plates were observed for a zone of inhibitions around the impregnated discs. Bald zone represented the inhibition of phosphorylation as spore or hyphae formation was halted in the absence of phosphorylation, while a clear zone of inhibition represented the potential killing effect of sample extract on test strain.

### 2.4. Data Analysis

All the assays were repeated in triplicate and presented as mean standard deviation. One way ANOVA and post-hoc Turkey HSD (Honest significant difference) test was employed for the comparison of mean values of results. SPSS (IBM SPSS Statistic 20) was used to determine the significance at *p* < 0.05 (95% confidence interval). Spearman correlation was found among phytochemicals and antioxidant activities. LC_50_ and IC_50_ values were calculated using table curve 2D version 4 software.

## 3. Results

### 3.1. Percent Extract Recovery

**Figure 1:**
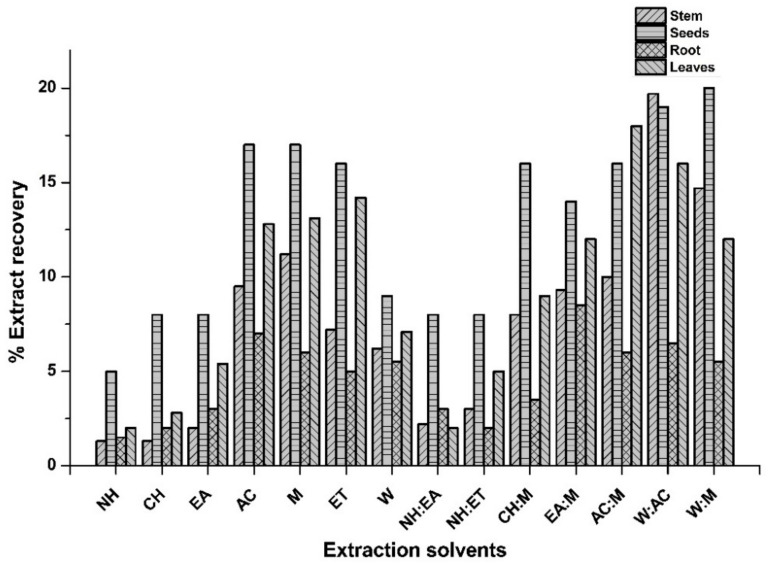
Percent extract recovery of stem, root, seeds, and leaves of *P. glabrum* using various individual solvents and their combination (1:1) for extraction. NH: *n*-hexane; CH: Chloroform; AC: Acetone; EA: Ethyl acetate; M: Methanol; ET: Ethanol; W: Distilled water; NH:EA: *n*-hexane + Ethyl acetate; NH:ET: *n*-hexane + Ethanol; CH:M: Chloroform + Methanol; EA:M: Ethyl acetate + Methanol; AC:M: Acetone + Methanol; W:AC: Distilled water + Acetone; W:M: Distilled water + Methanol.

Extraction is the first crucial step towards natural product drug discovery and involves the separation of active constituents from undesired inactive residues. A diversified range of solvent systems as well as sonication-aided maceration was used in order to achieve this target. Total 56 extracts of stem, roots, seeds, and leaves of *P. glabrum* were prepared in different solvent systems by the process of maceration and were then analyzed to explore various bioactive phytochemicals as well as biological activities. The maximum yield of extract was obtained by W:AC in stem, W:M in root, EA:M in seeds, and AC:M in leaves, i.e., 19.7%, 20.0%, 8.5%, and 18.0% *w*/*w*, respectively (Figure 1). The current assessment of extraction yield utilizing various solvents signifies that the solvent and sample composition are the most vital parameters that effect the extraction yield under constant conditions. A decreasing pattern was observed between the polarity of employed solvents and extraction yield as the yield was obtained with polar solvents systems, i.e., W:M and W:AC. The difference in extract recovery might be attributed to the varying polarity of solvents employed, which facilitated the extraction of secondary metabolites having diversified chemical composition [15]. Current findings were in agreement with the previous work where maximum extract yield was obtained when W:M was utilized as the extraction solvent [16].

### 3.2. Phytochemical Analysis

**Figure 2:**
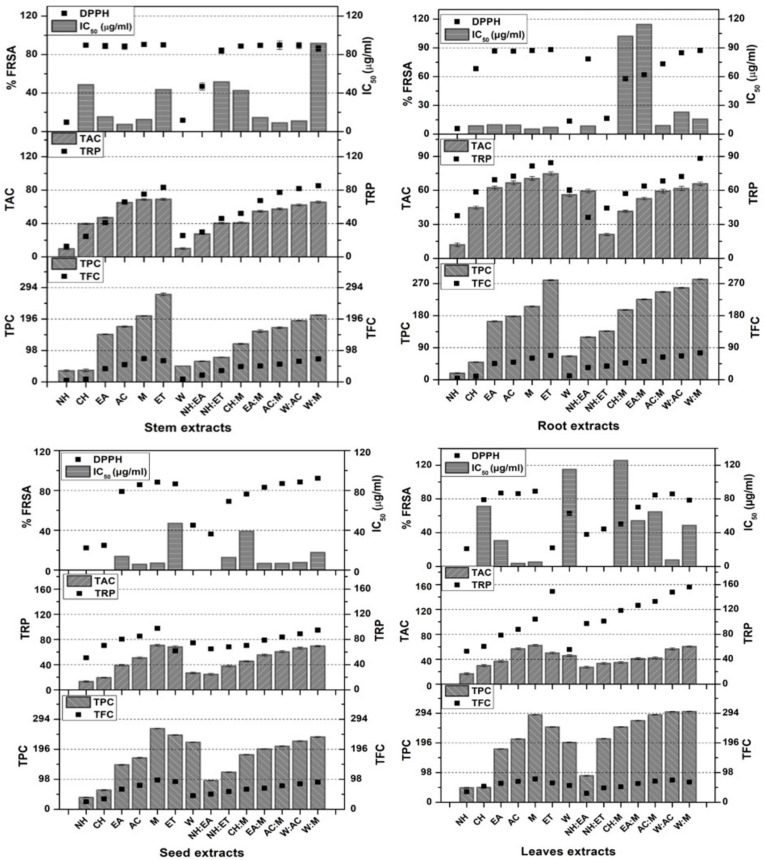
TPC (µg GAE/mg extract), TFC (µg QE/mg extract), TAC (µg AAE/mg extract), TRP (µg AAE/mg extract) and %FRSA (DPPH assay) of stem, root, seeds, and leaves of *P. glabrum* employing various solvents and their combinations. Values are presented as mean ± SD from triplicate investigations.

Phenolics and flavonoids are known for their antioxidant activity in biological systems due to their nascent oxygen and free radical quenching ability [17,18]. The methoxy, hydroxyl, and ketonic functional groups present in these compounds might contribute towards the antioxidant potential [19]. Lipid peroxidation inhibition and free radical scavenging are the most considerable traits which make these compounds pharmacologically active. Comparative analysis of results showed maximum phenolic contents in terms of gallic acid equivalents (µg GAE/mg extract) in W:M leaves extracts, i.e., 299.78 ± 0.89 µg GAE/mg extract. While a minimum of the TPC were quantified in NH root extracts 18.34 ± 1.63 µg GAE/mg extract (Figure 2). On the other hand, the results of TFC quantified in *P. glabrum* revealed that the highest TFC were found in seeds M extracts (95.66 ± 1.39 µg QE/mg extract), while the lowest flavonoid contents were quantified in NH roots extracts (4.25 ± 1.64 µg QE/mg extract) (Figure 2). A significant antioxidant activity of *P. glabrum* might be attributed to the presence of polyphenols and flavonoids or any other natural antioxidants. These results strongly correlate with the previous findings where phenolic substances were reported to have stupendous antioxidant activity [20].

HPLC-DAD fingerprinting of *P. glabrum* extracts in the different solvent systems was compared with the retention times and UV absorption spectra of various reference polyphenols, i.e., rutin, caffeic acid, gallic acid, quercetin, apigenin, kaempferol, catechin, and myricetin. The calibration curve equations of used standards along with the significance, LOD, and LOQ values are presented in Table 1. Based upon these parameters, samples were analyzed for the presence of respective polyphenols.

**Figure 3:**
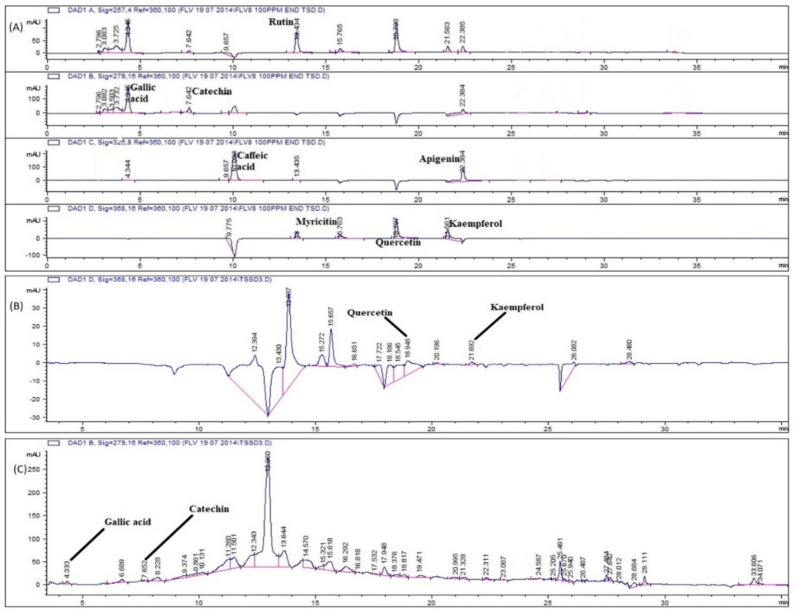
HPLC chromatograms of (**A**) standard polyphenols, (**B**,**C**) AC:M seeds extract of *P. glabrum*.

Significant polyphenols were detected in different parts of *P. glabrum* (Table 2). Different types of polyphenols, i.e., rutin, kaempferol, gallic acid, quercetin, apigenin, and catechin, were detected in most of the samples, as depicted in AC:M seed extract chromatogram (Figure 3). The results of polyphenol can be positively correlated with the phytochemical analysis and antioxidant activities. Our predicted correlation was also in agreement with the previous studies, where quercetin [21] and gallic acid [22] were found to have antioxidant potentials. In addition to these activities, the polyphenols also possess antimicrobial [22], anticancer [23], antidiabetic and antiadipogenic activities [24]. So it can also be depicted from the given data that antioxidant, antimicrobial and cytotoxic activities of *P. glabrum* may be due to the presence of these polyphenols, as it is evident that plant phenols are capable of inhibiting or attenuating the initiation, progression, and metastasis of cancer cells.

Solubility of polyphenols is mainly dependent upon the nature of the solvent used, degree of polymerization of polyphenols, as well as conjugation with other secondary metabolites, and formation of insoluble complexes [24]. The current study significantly highlights the impact of solvent variable polarities and plant parts on polyphenols content. Detection of apigenin in stem and leaves parts as well as rutin in EA and EA:M leaves extracts elaborate on the previous findings in which rutin and apigenin had been confirmed in M extract of whole *P. glabrum* plant by means of HPTLC analysis [25]. Therefore present results considerably show the presence of important polyphenols in individual plant parts to select the best plant part candidate for advanced exploration of pharmacological perspective.

### 3.3. Antioxidant Potential

The results of DPPH assay showed that the highest %FRSA was observed in M stem, ET root, W:M seeds, and M leaves extracts, i.e., IC_50_ values 12.75, <7.4, 17.75, and <7.4 µg/mL, respectively. (Figure 2). Twenty-nine out of 56 samples exhibited ≥80% FRSA with IC_50_ < 7.4 µg/mL suggesting that the plant has strong antioxidant potential. Our results of the antioxidant capabilities of the subject plant were comparable to the results of ascorbic acid (IC_50_ 14.56 µg/mL), which predicts that the plant has strong antioxidant potential.

Results of total antioxidant capacity assay of test samples of *P. glabrum* revealed that the highest TAC was observed in ET extracts of stem and root (69.01 ± 0.97 and 74.71 ± 1.76 µg AAE/mg) as well as M extracts of seeds and leaves (70.77 ± 1.63 and 62.31 ± 1.48 µg AAE/mg, respectively) (Figure 2).

**Figure 4:**
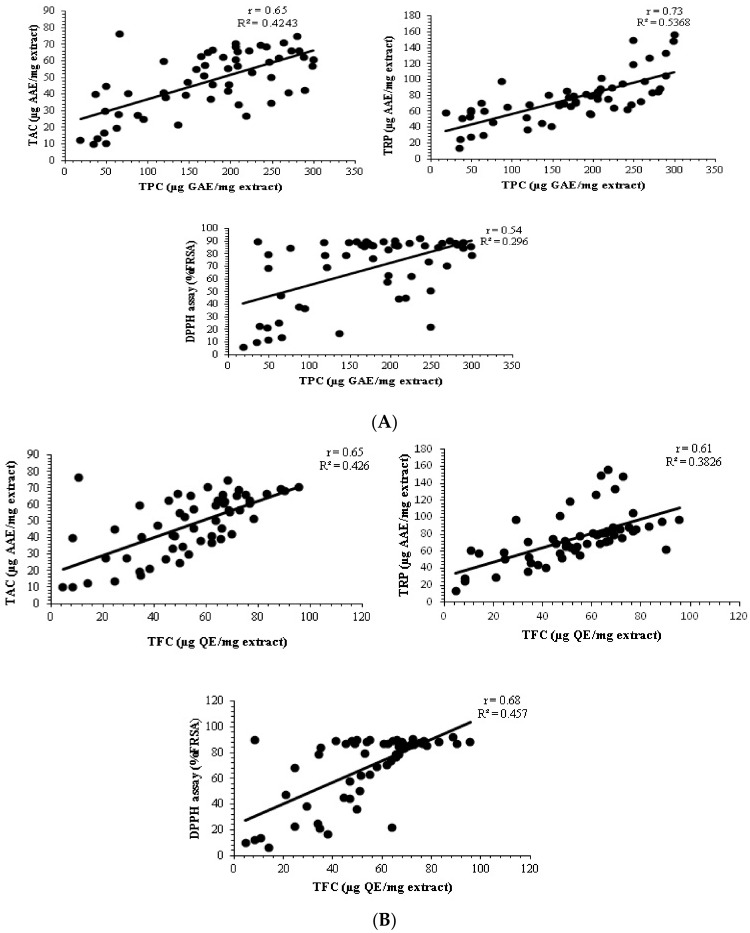
(**A**). Correlation between total phenolic content and different antioxidant assays. (**B**)**.** Correlation between total flavonoid content and different antioxidant assays.

The reducing power potential of *P. glabrum* crude extracts was expressed as µg AAE/mg extract. Highest TRP among all extracts was measured in W:M extracts of stem, roots, leaves (85.14 ± 1.13, 88.19 ± 0.67, and 156.00 ± 0.89 µg AAE/mg extract, respectively) and M extract of seeds (97.58 ± 1.67 µg AAE/mg extract) (Figure 2). Maximum antioxidant potentials were observed in M, ET, and aqueous organic solvent extracts (W:M and W:AC). Positive correlation (Spearman correlation) was observed between phytochemicals and antioxidant activities i.e., Spearman correlation coefficient (r) for TPC/TAC = 0.65, TPC/TRP = 0.73, TPC/DPPH = 0.54, TFC/TAC = 0.65, TFC/TRP = 0.61, and TFC/DPPH = 0.68 (Figure 4A,B). Specific determination of antioxidant capacity according to extraction solvent and plant parts suggests that polar leaves and seeds extracts of *P. glabrum* have maximum antioxidant potential, possibly due to the presence of polyphenols. Correlation analysis among phytochemicals and antioxidants with positive results indicates that the antioxidant activities enhance with an increase in phenolic and flavonoid contents. Thus, mechanism-based information was obtained from this study that phenols and flavonoids might exert their antioxidant effects by three methods, i.e., free radical scavenging, chelate formation, and complex formation. Our results positively correlate with the previous findings where the antioxidant potential of polyphenols was considered to be due to the same effects and mechanisms [24,26]. Natural antioxidants are helpful in preventing disease by quenching free radicals and blocking oxidation. They are also involved in ROS generation inhibition and adjustment of intracellular redox potential. It is also stated that there is an indirect link between antioxidant intake and the occurrence of human disease. The current study also emphasizes that increasing polarity increases the antioxidant activity, so ET and M, along with the aqueous organic extracts (W:M and W:AC), might be the best candidates for isolation of antioxidant compounds and can also be used in herbal therapeutic preparations.

### 3.4. Antimicrobial Assays

*P. glabrum* has also traditionally been used as an antimicrobial remedy against infectious diseases [27]. In natural extracts, antimicrobial activity may vary due to synergistic and antagonistic effects of the phytochemicals, as has previously been reported [22]. Both polar and nonpolar leaves and seeds extracts, i.e., EA, M, AC:M, EA, AC, CH:M, CH, and NH exhibited significant antibacterial activities against the tested strains. Maximum zones of inhibition were observed in the case of AC:M seeds and roots extracts against *S. typhimurium.* While in the case of roots, the maximum zone was observed for AC:M against *S. typhimurium* and *M. luteus.* Leaves extracts also exhibited varying degrees of antibacterial activity with a maximum zone of inhibition observed was by NH:EA against *S. typhimurium* (Table 3).

Extracts with significant results (zone of inhibition ≥ 10 mm) were further tested for MIC determination, and among all the samples, CH extract of seeds exhibited the least MIC value (3.7 µg/mL) against *S. typhimurium*. These results support the previous findings in which ethyl acetate and methanol fractions of leaves of *P. glabrum* showed moderate activity against *B. subtilis* and *P. vulgaris* [28]. Both results show a parallel relationship with each other in terms of solvent effect and plant part.

A varying, polarity-independent antifungal activity was also observed by the different extracts. Among all samples, leaves extracts were significantly effective against the tested antibacterial strains. In the case of stem, the maximum zone of inhibition was observed by NH:EA and NH against *F. solani* and *A. fumigatus* strains, respectively. No significant activity was observed against *Mucor* sp. In the case of root extracts, the highest inhibition potential was observed by W:M against *A. flavus, A. niger*, and *A. fumigatus*. Seed extracts did not exhibit significant antifungal activity demonstrating only NH extract to be moderately active against *A. flavus* (10 ± 1.00 mm). Maximum zone of inhibition against *A. niger* was observed for W, NH, and CH:M, while extracts were least effective against *A. fumigatus*, *Mucor* sp., and *F. solani*. In the case of leaves, maximum zone of inhibition was observed by AC:M, CH:M, NH:EA, and W, AC and CH against *A. flavus, A. niger*, *F. solani*, *A. fumigatus*, and *Mucor* sp., respectively (Table 4). Our work on the antifungal activity of *P. glabrum* is supported by another report in which the subject plant showed good activity against *Colletotrichum truncatum* [29]. Our results are contradicted to findings of another study involving different species of the same genus, in which methanol, hexane, aqueous, and petroleum ether extracts of whole *Polygonum equisetiforme* did not show any activity against ten bacterial strains and four fungal test species [30].

### 3.5. Enzyme Inhibition Assay

**Figure 5:**
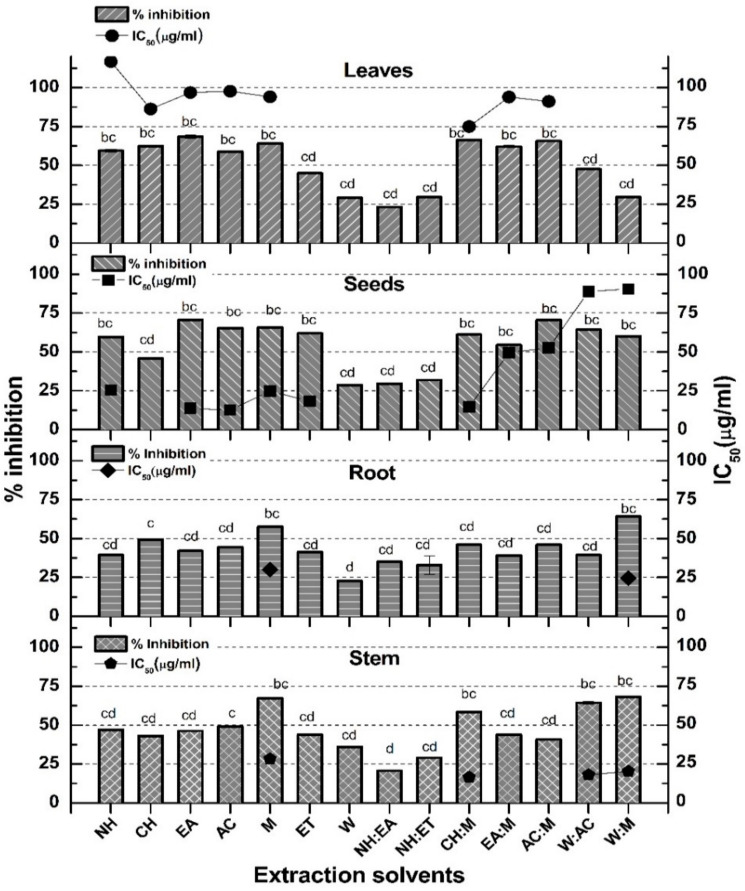
α-amylase inhibition by various solvent extracts of stem, root, seeds, and leaves of *P. glabrum*. The IC_50_ of acarbose (positive control) was 33.73 ± 0.12 µg/mL. The experiment was performed in triplicate, and values are presented as mean ± standard deviation. ^a−d^ means the difference is highly significant, slightly significant, and significant at *p* < 0.05.

The antidiabetic potential of the test extracts was assessed by α-amylase inhibition assay. The inhibitory enzyme activities in terms of %inhibition of different parts of *P. glabrum* are presented in Figure 5. Maximum enzyme inhibition was observed by W:M stem (66.36% ± 0.06%, IC_50_ 20.34 µg/mL) as well as root extract (64.11% ± 0.12%, IC_50_ 24.59 µg/mL), EA seeds (70.66% ± 0.19%, IC_50_ 13.66 µg/mL) and leaves extract (68.45% ± 0.1%, IC_50_ 96.88 µg/mL). The least activity was observed in the case of NH test samples of all parts (Figure 5). A positive correlation (r = 0.592 at *p* = 0.01) was observed between flavonoid contents and α-amylase inhibiting activity. The α-amylase inhibition activity showed a mild positive correlation with the flavonoids (r = 0.5934). In the current report, the α-amylase inhibition method was used for the determination of in vitro antidiabetic potential, as it is considered to be an effective tool to keep glucose levels in the permissible range. The α-amylase enzyme catalyzes the hydrolysis of starch, glycogen, and various oligosaccharides at α-1,4-glycosidic linkages by converting them into disaccharides which are further simplified into simpler sugars by α-glucosidase, making it readily available for the intestinal absorption. So enzymatic control at the intestinal level is an important strategy to diminish the absorption of glucose for the control of diabetes [31]. Therefore, a long-awaited effort is in the process of searching for effective and nontoxic inhibitors of α-glucosidase and α-amylase. The results displayed that M, AC:M, and W:M exhibited the highest inhibitory activity while it was moderate in nonpolar extracts (AC, EA and their combinations with M). The correlation value between flavonoids and α-amylase inhibition was less significant (r = 0.5934), which contradicts the previous findings where flavonoids were found to be responsible for α-amylase inhibition [32].

### 3.6. Cytotoxicity Assays

**Figure 6:**
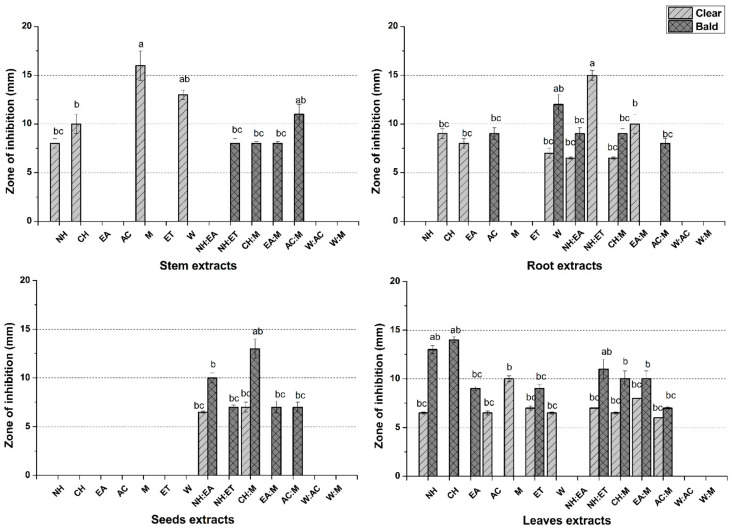
Protein kinase inhibitory activities of various solvent extracts of stem, seeds, leaves, and roots of *P. glabrum*. Positive control (surfactin at 20 µg/disc) gave 33 ± 1.10 mm bald zone of inhibition. Values (mean ± SD) are the average of triplicate investigations. ^a−d^ means the difference is highly significant, slightly significant, and significant at *p* < 0.05.

In protein kinase inhibition assay, significant bald zones were observed by AC:M stem extract (11 ± 1.15 mm), W root extract (12 ± 1.20 mm), CH:M seeds extract (13 ± 0.58 mm), and CH leaves extract (14 ± 1.13 mm). Whereas NH:ET root, M stem, and leaves extracts showed noteworthy clear zones, i.e., 15 ± 1.53, 16 ± 1.53, and 10 ± 1.00 mm. No significant clear zone was observed by seed extracts (Figure 6). Remarkable results by W:AC of stem, W of root, and NH:EA of seed, and most prominently CH of leaves proposed that these extracts would be appropriate for the isolation of bioactive constituents of *P. glabrum* that may assist in developing a promising kinase inhibitory drug. No polarity-based activity trend of protein kinase inhibition potential was observed in *P. glabrum* crude extracts while plant part-dependent activity was observed. In the past few decades, there has been a noteworthy need for the development of safe and cost-effective protein kinase inhibitors from plants [33]. Phosphorylation at tyrosine and serine/threonine residues of cells regulates major important cascades involved in various biological processes ranging from cell proliferation to apoptosis [33]. Deregulated or uncontrolled phosphorylation by protein kinases at serine/threonine and tyrosine residues by genetic modifications in cells leads to the onset of cancer. So, targeting those cascades of kinases has developed to be a remarkable objective for the isolation of anticancer compounds [34]. So far, 500 kinases have been identified in the human genome, and the allosteric binding of extracts with one of these active or inactive kinases will be vital in the identification and development of anticancer compounds [35]. The kinases are employed by the *Streptomyces* species for the development of the aerial hyphae; thus, the inhibition is considered as a marker of protein kinase inhibitory potential [13]. This requirement had been utilized in the current study to determine the kinase inhibitory profile of the given crude samples of *P. glabrum* in a bio prospective manner so that their anticancer potential could be evaluated. Positive results might be attributed to the presence of gallic acid (W:AC of stem) and/or apigenin (W of root) according to a previous finding in which gallic acids and other polyphenols led to inhibition of phosphorylation of protein kinases [36].

The cytotoxicity potential of the investigational plant *P. glabrum* test samples was also evaluated by brine shrimp lethality activity. This assay has long been used as a valuable biological probe for determining cytotoxic profiles of test extracts [37]. Brine shrimps (*Artemia salina*) larvae have been considered as a simple and efficient tool for screening of antitumor, insecticidal, antimicrobial, and antimalarial activities of samples [38,39]. It is suggested that the shrimp larvae behave like the mammalian carcinoma cells, and the anticancer effects of tested samples (crude extracts) might well elucidate their antitumor and cytotoxic activity [38]. Most significant cytotoxicity was seen in nonpolar extracts, i.e., CH, NH, NH:EA, while crude extracts of polar solvents were comparatively less cytotoxic, having higher LC_50_ values. The activity was found to be concentration-dependent, i.e., decreasing test sample concentration decreased %mortality (Table 5). The results are in strong agreement with previously published reports against brine shrimps [13]. The LC_50_ of most of the samples was less than 250 µg/mL, which signifies a noticeable cytotoxic profile of test extracts. The acquired cytotoxic potential of the plant might be attributed to the presence of a high quantity of secondary metabolites, e.g., phenols, flavonoids, alkaloids, etc. [39]. Furthermore, a previous study also claimed that *P. glabrum* roots extracts were nontoxic and safe at a dose of 2 g/Kg in rat models of acute and chronic toxicity [40]. Screening of the crude test samples with minimum LC_50_ values and higher safety profiles might offer valuable cytotoxic and/or antitumor secondary metabolites. Thus, this pilot study results would offer an ideal pathway for further screening and isolation of potentially important secondary metabolites.

## 4. Conclusions

In the current report, various phytochemical, antioxidant, antimicrobial, antidiabetic, and cytotoxic assays have been performed on stem, roots, seeds, and leaves of *P. glabrum* to corroborate its folklore utilization via modern scientific intervention. A wide range of polarity solvents led to the formation of extracts with varied pharmacological profiling. The current study proposes that extracts in the polar solvents showed superior antioxidant, moderately polar extracts gave better antimicrobial activity while lesser polar solvents led to the extracts with significant cytotoxicity profile. Thorough screening suggests that polarity of solvent and part of the plant used to decide the fate of biological efficacy, nature of the pharmacological response, and extraction efficiency. It also highlights the need for expansion of our pilot study results and critical evaluation to initiate bioactivity guided isolation from this stupendous medicinal plant.

## Figures and Tables

**Table 1:** Calibration curve equations, retention times, correlation coefficient, LOD, and LOQ values of all the standards.

S. No.	Standards	Retention Time-RT (min)	Calibration Curve Equation	Correlation Coefficient (r2)	LOD (μg/mL)	LOQ (μg/mL)
**1**	Gallic acid	4.34	y = 24.857x − 45.174	0.9979	7.2	21.9
**2**	Catechin	7.64	y = 7.985x − 17.565	0.9995	3.5	10.7
**3**	Caffeic acid	10.09	y = 26.097x + 95.435	0.9924	13.7	41.4
**4**	Rutin	13.43	y = 8.336x + 22.217	0.9966	9.9	27.8
**5**	Myricetin	15.76	y = 5.227x − 6.304	0.9988	5.4	16.3
**6**	Quercetin	18.80	y = 12.210x − 20.348	0.9978	7.3	22.2
**7**	Kaempferol	21.56	y = 9.994x + 15.261	0.9998	2.3	7.0
**8**	Apigenin	22.38	y = 18.111x + 25.565	0.997	5.0	15.2

**Table 2:** Polyphenols identified in various solvent extracts of stem, root, seeds, and leaves of *P. glabrum* by HPLC-DAD analysis.

Extract	Polyphenols (µg/mg Extract)					
	Gallic Acid	Rutin	Catechin	Apigenin	Quercetin	Kaempferol
Stem						
**EA**	0.43 ± 0.01 **	-	-	0.10 ± 0.00 ***	-	-
**AC**	-	-	-	0.07 ± 0.00	-	-
**M**	0.22 ± 0.01 ***	-	-	0.09 ± 0.00	-	-
**ET**	0.46 ± 0.01**	-	-	0.11 ± 0.01 ***	-	-
**W**	0.12 ± 0.03 ***	-	-	-	-	-
**EA:M**	0.26 ± 0.01 ***	-	-	1.26 ± 0.02 *	-	-
**AC:M**	0.44 ± 0.03 **	-	-	-	-	-
**W:AC**	0.07 ± 0.00	-	-	1.10 ± 0.02 *	-	-
**W:M**	0.10 ± 0.02 ***	-	-	-	-	-
**Root**						
**EA**	0.40 ± 0.03 **	-	-	-	-	-
**M**	0.44 ± 0.02 **	-	-	-	-	-
**ET**	0.51 ± 0.01	-	-	-	-	-
**W**	0.2 ± 0.03 ***	-	-	-	-	-
**EA:M**	0.46 ± 0.02 **	-	-	-	-	-
**AC:M**	0.16 ± 0.01 ***	-	-	-	-	-
**W:AC**	0.09 ± 0.00	-	-	-	-	-
**W:M**	0.13 ± 0.05 ***		-	-	-	-
**Seed**						
**EA**	-	-	0.98 ± 0.10 *	-	-	-
**AC**	-	-	0.93 ± 0.30 *	-	-	-
**M**	0.40 ± 0.10 **	-	0.81 ± 0.21 *	-	1.30 ± 0.50 *	0.59 ± 0.05 **
**ET**	0.46 ± 0.10 **	-	2.06 ± 0.32 *	-	-	-
**W**	0.18 ± 0.05 ***	-	-	-	-	-
**EA:M**	0.19 ± 0.03 ***	-	1.87 ± 0.1 1*	-	3.28 ± 0.10 *	0.52 ± 0.30 **
**AC:M**	0.42 ± 0.05 **	-	2.55 ± 0.10 *	-	3.10 ± 0.11 *	0.76 ± 0.10 **
**W:AC**	0.10 ± 0.00	-	1.02 ± 0.10 *	-	0.7 ± 0.05 **	0.09 ± 0.00
**W:M**	0.21 ± 0.02 ***	-	-	-	-	-
**Leaves**						
**EA**	0.09 ± 0.00	0.21 ± 0.03 ***	-	0.8 ± 0.05 *	0.5 ± 0.02 **	1.6 ± 0.04 *
**AC**	0.06 ± 0.01	-	-	-	-	1.21 ± 0.05 *
**M**	0.09 ± 0.00	-	-	-	-	0.5 ± 0.05 **
**ET**	0.25 ± 0.02 ***	-	-	-	-	1.36 ± 0.10 *
**W**	-	-	-	-	-	-
**EA:M**	0.43 ± 0.10 **	0.15 ± 0.05 ***	-	1.45 ± 0.02 *	0.39 ± 0.03 **	2.71 ± 0.04 *
**AC:M**	0.26 ± 0.05 ***	-	-	0.06 ± 0.00	0.27 ± 0.01 **	2.29 ± 0.05 *
**W:AC**	0.03 ± 0.00	-	-	0.92 ± 0.04 *	-	-
**W:M**	0.06 ± 0.01	-	-	-	-	-

- = Not detected; no polyphenol was detected in NH, CH, NH:EA, NH:ET, and CH:M of the given parts of *P. glabrum*. Myricetin and Caffeic acid were not identified in any of the samples. Means difference is highly significant (*), slightly significant (**), significant (***) at *p* < 0.05. Values are presented as mean ± standard deviation from triplicate investigations.

**Table 3:** Results of antibacterial activity of different solvent extracts of stem, root, seeds, and leaves of *P. glabrum*.

Extract	Diameter of Growth Inhibition Zone (mm ± SD) at 100 µg/disc, MIC (µg/mL)							
	Gram-Negative				Gram-Positive			
	*K. pneumoniae*	MIC	*S. typhimurium*	MIC	*M. luteus*	MIC	*S. aureus*	MIC
Stem								
**NH**	10 ± 0.58 **	100	9 ± 0.00 **	100	7 ± 0.58	>100	9 ± 0.76 **	>100
**CH**	8 ± 0.58	>100	7 ± 0.58	>100	8 ± 0.58 ***	>100	8 ± 0.76	>100
**EA**	9 ± 0.58 **	>100	9 ± 0.00 **	>100	11 ± 0.00 **	33.3	9 ± 0.50	>100
**AC**	9 ± 0.29 **	>100	10 ± 0.58 **	33.3	11 ± 0.58 **	100	-	>100
**M**	9 ± 0.58 **	>100	8 ± 0.58 ***	100	7 ± 0.58	>100	10 ± 0.58 **	100
**ET**	8 ± 0.58	>100	7 ± 1.00	>100	10 ± 0.58 **	100	-	>100
**W**	8.5 ± 0.50 ***	>100	9 ± 0.58 **	>100	6 ± 0.00	100	8 ± 0.58 ***	>100
**NH:EA**	10 ± 0.58 **	100	11 ± 0.58 **	33.3	9 ± 0.58 **	>100	8 ± 0.58 ***	>100
**NH:ET**	10 ± 0.58 **	100	11 ± 1.00 **	33.3	7 ± 0.00 ***	>100	9 ± 0.58 **	>100
**CH:M**	8 ± 0.58	>100	-	>100	9 ± 0.58 **	>100	8 ± 1.00	>100
**EA:M**	11 ± 0.58 **	33.3	13 ± 0.58 *	33.3	8 ± 0.00	>100	8 ± 0.58	>100
**AC:M**	10 ± 0.58 **	100	14 ± 0.58 *	11.1	8 ± 0.58 ***	>100	7 ± 0.58 ***	>100
**W:AC**	8 ± 0.58 ***	100	10 ± 0.58 **	33.3	-	>100	8 ± 0.29	>100
**W:M**	8 ± 0.58 ***	>100	10 ± 1.15 **	33.3	9 ± 0.58 **	>100	9 ± 0.00 **	>100
**Root**								
**NH**	10 ± 0.58 **	100	9 ± 0.58	>100	7 ± 0.58	>100	8 ± 0.58 ***	>100
**CH**	10 ± 0.58 **	100	10 ± 0.58 **	100	9 ± 0.00 **	>100	7 ± 1.00	>100
**EA**	10 ± 0.58 **	100	11 ± 0.58 **	33.3	9 ± 0.58 **	>100	8 ± 0.58 ***	>100
**AC**	10 ± 0.00 **	33.3	11 ± 0.58 **	33.3	9 ± 0.58 **	>100	6 ± 0.00	>100
**M**	11 ± 0.58 **	33.3	13 ± 0.58 *	33.3	9 ± 0.58 **	>100	7 ± 1.00 ***	>100
**ET**	12 ± 0.29 **	100	11 ± 0.58 **	94	8 ± 0.00 ***	>100	9 ± 1.15 **	>100
**W**	7 ± 0.58	>100	10 ± 0.58	100	7 ± 0.58	>100	7 ± 1.00	>100
**NH:EA**	7 ± 0.58 ***	>100	13 ± 0.58 *	33.3	10 ± 0.58 **	100	8 ± 0.58 ***	>100
**NH:ET**	7 ± 0.58	>100	14 ± 0.58 *	11.1	-	>100	8 ± 0.58	>100
**CH:M**	9 ± 1.73 **	>100	14 ± 0.58 *	11.1	11 ± 0.00 **	100	8 ± 1.00 ***	>100
**EA:M**	8 ± 0.58 ***	>100	14 ± 1.00 *	11.1	8 ± 0.58	>100	8 ± 0.58 ***	>100
**AC:M**	7 ± 0.58 ***	>100	14 ± 1.15*	33.3	14 ± 0.58 *	11.1	8 ± 0.58	>100
**W:AC**	7 ± 0.58 ***	>100	13 ± 0.58 *	33.3	10 ± 0.58 **	100	9 ± 0.58 **	>100
**W:M**	7 ± 0.00	>100	10 ± 0.58 **	100	10 ± 0.58 **	100	8 ± 0.58 ***	>100
**Seeds**								
**NH**	10 ± 0.58	100	13 ± 0.58 *	11.1	9 ± 0.58	>100	9 ± 0.58	>100
**CH**	10 ± 0.58 **	100	20 ± 1.00 *	3.7	9 ± 0.58 **	>100	9 ± 0.58 **	>100
**EA**	7 ± 0.58 ***	>100	14 ± 1.00 *	4.7	10 ± 0.00 **	100	8 ± 1.00 ***	>100
**AC**	9 ± 0.58 **	>100	9 ± 1.00	>100	10 ± 0.58 **	100	6 ± 0.58 ***	>100
**M**	10 ± 0.58	100	16 ± 0.58 *	3.7	7 ± 0.58	>100	9 ± 0.00 **	>100
**ET**	9 ± 0.58 **	>100	14 ± 0.58 *	11.1	10 ± 0.58 **	100	8 ± 0.58	>100
**W**	10 ± 0.58 **	100	7 ± 0.58	>100	7 ± 0.00 ***	>100	7 ± 0.58	>100
**NH:EA**	12 ± 0.58 **	33.3	10 ± 0.58 **	100	9 ± 0.58 **	>100	9 ± 0.58 **	>100
**NH:ET**	10 ± 0.58 **	100	12 ± 1.00 **	33.3	8 ± 0.58	>100	10 ± 0.58 **	100
**CH:M**	9 ± 0.58 **	>100	15 ± 0.58 *	3.7	9 ± 0.00 **	>100	10 ± 0.58 **	100
**EA:M**	11 ± 0.58 **	100	10 ± 0.58 **	100	8 ± 0.00	>100	9 ± 0.00 **	>100
**AC:M**	9 ± 0.55	>100	12 ± 0.58 **	33.3	8 ± 0.58 ***	>100	8 ± 1.00	>100
**W:AC**	9 ± 1.15**	>100	9 ± 1.00 **	>100	7 ± 0.58	>100	9 ± 1.00 **	>100
**W:M**	9 ± 0.58 **	>100	8 ± 0.58	>100	9 ± 0.58 **	>100	8 ± 0.58	>100
**Leaves**								
**NH**	9 ± 0.58 **	>100	9 ± 0.58	>100	9 ± 0.58 **	>100	10 ± 0.58 **	100
**CH**	8 ± 0.58	>100	15 ± 0.58 *	3.7	9 ± 0.00	>100	8 ± 0.58	>100
**EA**	10 ± 0.58 **	100	10 ± 1.53**	100	10 ± 0.58 **	100	8 ± 0.58 ***	>100
**AC**	10 ± 0.58 **	100	12 ± 0.58 **	33.3	-	>100	10 ± 0.58 **	100
**M**	10 ± 0.58 **	100	10 ± 0.58	100	9 ± 0.58 **	>100	8 ± 0.58	>100
**ET**	9 ± 0.58	>100	8 ± 0.58 ***	>100	8 ± 0.58 ***	>100	8 ± 0.58 ***	>100
**W**	9 ± 0.58 **	>100	11 ± 0.58 **	100	-	>100	8 ± 1.15 ***	>100
**NH:EA**	9 ± 0.58 **	>100	16 ± 0.58 *	3.7	8 ± 0.58	>100	8 ± 0.58	>100
**NH:ET**	10 ± 0.58 **	100	8 ± 0.58	>100	8 ± 0.58 ***	>100	9 ± 1.00 **	>100
**CH:M**	8 ± 0.58	>100	10 ± 1.00 **	100	6 ± 0.00	>100	8 ± 0.58 ***	>100
**EA:M**	7 ± 0.58 ***	>100	10 ± 0.58 **	100	11 ± 0.58 **	100	10 ± 0.58 **	100
**AC:M**	8 ± 0.58	>100	10 ± 1.15**	100	7 ± 0.58 ***	>100	8 ± 0.58	>100
**W:AC**	12 ± 0.58 **	33.3	10 ± 1.15**	100	6 ± 0.00	>100	8 ± 0.58	>100
**W:M**	10 ± 0.58	100	10 ± 0.58 **	100	7 ± 0.00 ***	>100	8 ± 0.58 ***	>100
**Standards**								
**Ciprofloxacin**	17 ± 1.6	0.06	10 ± 0.07	0.06	24 ± 0.95	0.8	15 ± 0.85	0.125
**Cefixime**	19.5 ± 1.3	0.2	21 ± 0.85	0.02	24.6 ± 0.6	0.8	22.5 ± 0.11	0.25

Zones of inhibition include the diameter of disc (6 mm). Each sample concentration was 100 µg per disc (5 µL). Values (mean ± SD) are the average each plant extract analyzed individually in triplicate (n = 1 × 3). - = No activity in disc diffusion assay. Positive control (cefixime and ciprofloxacin) concentration was 20 µg/disc. Negative control (DMSO) was inactive against each bacterial strain. Means difference is highly significant (*), slightly significant (**), and significant (***) at *p* < 0.

**Table 4:** Results of antifungal activity of different solvent extracts of stem, root, seeds, and leaves of *P. glabrum*.

Extract	Diameter of Growth Inhibition Zone (mm) at 100 µg/disc, MIC (µg/disc)									
	*A. flavus*	MIC	*A. niger*	MIC	*F. solani*	MIC	*A. fumigatus*	MIC	*Mucor* sp.	MIC
Stem										
**NH**	9 ± 1.00 **	-	7 ± 0.00 ***	-	9 ± 0.00 **	-	13 ± 0.58 *	100	7 ± 0.58	-
**CH**	8 ± 0.58	-	-	-	10 ± 0.58 **	-	7 ± 0.00	-	-	-
**EA**	7 ± 0.58 ***	-	7 ± 0.58 ***	-	-	-	8 ± 0.58 ***	-	-	-
**AC**	-	-	9 ± 0.58 **	-	8 ± 0.58 ***	-	13 ± 0.58 *	100	-	-
**M**	-	-	-	-	-	-	11 ± 0.58 **	-	-	-
**ET**	-	-	-	-	8 ± 0.58	-	8 ± 1.00	-	-	-
**W**	10 ± 1.73 **	-	8 ± 0.58 ***	-	9 ± 0.58 **	-	13 ± 1.52 *	100	7 ± 1.00 ***	-
**NH:EA**	9 ± 1.73 **	-	-	-	13 ± 0.58 *	100	-	-	-	-
**NH:ET**	-	-	11 ± 0.00 **	-	12 ± 0.58 *	100	7 ± 0.58	-	-	-
**CH:M**	8 ± 0.00 ***	-	-	-	-	-	-	-	-	-
**EA:M**	8 ± 1.15 ***	-	-	-	7 ± 0.00 ***	-	9 ± 1.15 **	-	-	-
**AC:M**	-	-	-	-	8 ± 0.00	-	-	-	-	-
**W:AC**	11 ± 1.00 **	-	-	-	-	-	12 ± 1.00 *	100	-	-
**W:M**	9 ± 0.58 **	-	-	-	-	-	12 ± 1.52 *	100	-	-
**Root**										
**NH**	8.5 ± 0.5	-	-	-	6.5 ± 0.00	-	9 ± 1.73 **	-	6.5 ± 0.50	-
**CH**	8 ± 1.52 ***		-	-	7 ± 0.58 ***	-	7 ± 0.58 ***	-	8 ± 0.58 ***	-
**EA**	-	-	-	-	8 ± 0.58	-	8 ± 1.15	-	-	-
**AC**	-	-	-	-	8 ± 0.58 ***	-	9 ± 1.52 **	-	8 ± 0.58 ***	-
**M**	10 ± 0.58 **	-	-	-	8 ± 0.58 ***	-	-	-	-	-
**ET**	-	-	-	-	8 ± 0.00	-	10 ± 1.52 **	-	-	-
**W**	9 ± 0.58 **	-	-	-	6.5 ± 0.02	-	9 ± 0.58 **	-	-	-
**NH:EA**	10 ± 0.58 **	-	7 ± 0.58	-	9 ± 0.58 **	-	12 ± 0.58 *	100	-	-
**NH:ET**	8 ± 1.15	-	-	-	8 ± 0.58 ***	-	6 ± 0.00	-	-	-
**CH:M**	9 ± 1.15 **	-	8 ± 0.58 ***	-	7 ± 0.58 ***	-	7 ± 0.58	-	-	-
**EA:M**	-	-	7 ± 0.00 ***	-	7 ± 0.00	-	11 ± 0.58 **	-	12 ± 0.58 *	100
**AC:M**	-	-	11 ± 0.58 **	-	8 ± 0.15 ***	-	12 ± 1.15 *	100	-	-
**W:AC**	10 ± 1.15 **	-	9 ± 0.58	-	12 ± 0.58 *	100	12 ± 0.00 *	100	12 ± 0.58 *	100
**W:M**	13 ± 1.73 *	100	10 ± 0.58 **	-	8 ± 0.58 ***	-	13 ± 0.58 *	100	10 ± 1.15 **	-
**Seed**										
**NH**	10 ± 1.00 **	-	11 ± 0.58 **	-	-	-	-	-	-	-
**CH**	-	-	9 ± 0.58 **	-	-	-	-	-	-	-
**EA**	-	-	8 ± 0.58	-	-	-	-	-	-	-
**AC**	-	-	8 ± 0.00 ***	-	-	-	-	-	-	-
**M**	-	-	9 ± 0.58 **	-	-	-	-	-	-	-
**ET**	-	-	8 ± 0.58	-	-	-	-	-	-	-
**W**	-	-	11 ± 1.67 **	-	-	-	-	-	-	-
**NH:EA**	-	-	9 ± 1.00 **	-	-	-	7 ± 0.58	-	-	-
**NH:ET**	-	-	8 ± 0.00	-	-	-	-	-	-	-
**CH:M**	-	-	11 ± 0.58 **	-	-	-	7 ± 0.58 ***	-	-	-
**EA:M**	-	-	8 ± 0.58	-	-	-	-	-	-	-
**AC:M**	-	-	7 ± 0.00 ***	-	-	-	-	-	-	-
**W:AC**	-	-	10 ± 0.58 **	-	-	-	-	-	-	-
**W:M**	-	-	11 ± 1.15	-	-	-	-	-	-	-
**Leaves**										
**NH**	8 ± 1.15 ***	-	10 ± 0.58 **	-	8 ± 0.58	-	10 ± 0.58 **	-	10 ± 1.52 **	-
**CH**	9 ± 0.58 **	-	9 ± 0.00 **	-	9 ± 0.58 **	-	11 ± 0.58 **	-	15 ± 1.00 *	100
**EA**	9 ± 1.73**	-	9 ± 0.00	-	8 ± 0.58 ***	-	10 ± 0.58	-	12 ± 0.58 *	100
**AC**	9 ± 0.58 **	-	7 ± 1.00	-	10 ± 0.00	-	9 ± 0.58 **	-	8 ± 0.58	-
**M**	8 ± 1.15	-	10 ± 1.53 **	-	9 ± 0.58 **	-	9 ± 0.58 **	-	9 ± 0.58 **	-
**ET**	10 ± 1.52 **	-	9 ± 0.00 **	-	9 ± 0.15 **	-	9 ± 0.58 **	-	11 ± 0.58	-
**W**	7 ± 0.58	-	7 ± 1.53	-	11 ± 0.58	-	9 ± 1.15**	-	9 ± 0.58 **	-
**NH:EA**	9 ± 0.58 **	-	10 ± 0.58 **	-	11 ± 0.58 **	-	9 ± 1.15	-	8 ± 0.58	-
**NH:ET**	9 ± 0.58	-	7 ± 0.58 ***	-	9 ± 0.58 **	-	10 ± 0.58 **	-	13 ± 0.58 *	100
**CH:M**	11 ± 0.58 **	-	7 ± 1.15	-	10 ± 0.58 **	-	9 ± 0.58 **	-	9 ± 0.58 **	-
**EA:M**	10 ± 0.58 **	-	8 ± 1.15***	-	11 ± 0.58	-	-	-	10 ± 1.53**	-
**AC:M**	9 ± 0.58 **	-	9 ± 0.58 **	-	11 ± 1.15 **	-	-	-	11 ± 0.58	-
**W:AC**	9 ± 0.00 **	-	10 ± 1.52 **	-	8 ± 0.58	-	7 ± 0.58	-	11 ± 0.58 **	-
**W:M**	7 ± 0.00	-	11 ± 1.52 **	-	8 ± 0.58 ***	-	9 ± 0.58 **	-	6.5 ± 0.50 ***	-
**Standard**										
**Clotrimazole**	30 ± 0.58	1.11	35 ± 2.30	1.11	28 ± 0.00	1.11	34 ± 0.58	1.11	31 ± 1.00	1.11

Zones of inhibition include the diameter of disc (6 mm). Each sample concentration was 100 µg per disc (5 µL). Values (mean ± SD) are the average of each plant extract analyzed individually in triplicate (n = 1 × 3). - = No activity in disc diffusion assay. Positive control (clotrimazole) concentration was 20 µg/disc. Negative control (DMSO) was inactive against each fungal strain. Means difference is highly significant (*), slightly significant (**), and significant (***) at *p* < 0.

**Table 5:** Brine shrimp lethality assay of various solvent extracts of stem, root, seeds, and leaves of *P. glabrum*.

Extracts	Percent Mortality at Various Concentrations (µg/mL)					
	500	250	125	63	32	LC_50_ (µg/mL)
**Stem**						
**NH**	53 ± 0.58	34 ± 1.15	-	-	-	351
**CH**	42 ± 0.58	41 ± 1.00	-	-	-	>500
**EA**	41 ± 0.58	31 ± 1.15	-	-	-	>500
**AC**	76 ± 1.53	54 ± 1.73	27 ± 1.53	20 ± 1.53	17 ± 1.15	200
**M**	69 ± 1.15	40 ± 1.00	-	-	-	300
**ET**	74 ± 1.15	65 ± 1.53	33 ± 1.53	25 ± 2.52	4 ± 1.53	248
**W**	25 ± 0.58	14 ± 1.00	-	-	-	>500
**NH:EA**	20 ± 0.58	20 ± 1.53	-	-	-	>500
**NH:ET**	50 ± 0.00	44 ± 0.58	-	-	-	>500
**CH:M**	54 ± 1.53	36 ± 0.53	-	-	-	405
**EA:M**	69 ± 1.00	61 ± 1.00	55 ± 2.00	40 ± 2.00	30 ± 2.00	100
**AC:M**	62 ± 0.58	55 ± 0.58	60 ± 1.53	31 ± 3.61	13 ± 2.52	81
**W:AC**	31 ± 1.15	11 ± 0.58	-	-	-	>500
**W:M**	30 ± 1.00	25 ± 1.00	-	-	-	>500
**Root**						
**NH**	72 ± 0.58	31 ± 1.00	-	-	-	347
**CH**	21 ± 0.58	20 ± 0.58	-	-	-	>500
**EA**	95 ± 1.53	83 ± 1.53	49 ± 1.15	41 ± 3.61	23 ± 1.53	130
**AC**	89 ± 1.00	78 ± 10.9	89 ± 1.15	84 ± 3.61	43 ± 1.15	40
**M**	75 ± 1.15	61 ± 0.00	43 ± 2.08	32 ± 2.00	13 ± 2.65	180
**ET**	74 ± 1.53	70 ± 0.58	62 ± 2.52	51 ± 3.21	32 ± 2.52	65
**W**	75 ± 1.15	61 ± 1.15	23 ± 2.08	13 ± 2.52	5 ± 0.58	60
**NH:EA**	66 ± 0.58	63 ± 1.00	34 ± 2.08	22 ± 2.65	9 ± 1.00	200
**NH:ET**	51 ± 0.58	31 ± 1.53	-	-	-	500
**CH:M**	82 ± 1.00	75 ± 1.00	18 ± 2.00	15 ± 2.00	9 ± 1.15	180
**EA:M**	80 ± 1.00	73 ± 1.53	67 ± 3.00	39 ± 2.08	9 ± 1.00	78
**AC:M**	87 ± 0.58	56 ± 1.00	50 ± 0.58	22 ± 2.52	20 ± 0.58	125
**W:AC**	29 ± 1.00	14 ± 1.00	-	-	-	>500
**W:M**	24 ± 1.00	14 ± 1.15	-	-	-	>500
**Seeds**						
**NH**	88 ± 1.00	85 ± 0.58	77 ± 3.51	68 ± 2.52	37 ± 2.52	43
**CH**	64 ± 1.00	48 ± 0.00	-	-	-	261
**EA**	71 ± 1.00	64 ± 1.15	52 ± 1.53	28 ± 2.00	19 ± 1.53	110
**AC**	83 ± 0.58	67 ± 0.58	53 ± 2.52	31 ± 9.87	28 ± 2.52	107
**M**	52 ± 1.53	43 ± 1.53	-	-	-	500
**ET**	62 ± 0.58	44 ± 1.73	-	-	-	289
**W**	20 ± 0.58	16 ± 1.00	-	-	-	>500
**NH:EA**	97 ± 0.58	89 ± 1.73	93 ± 2.52	78 ± 2.08	28 ± 2.65	51
**NH:ET**	47 ± 1.53	44 ± 1.15	-	-	-	>500
**CH:M**	64 ± 0.58	32 ± 0.58	-	-	-	310
**EA:M**	55 ± 0.58	54 ± 1.15	-	-	-	300
**AC:M**	51 ± 1.00	40 ± 0.58	-	-	-	500
**W:AC**	55 ± 0.58	47 ± 1.15	-	-	-	333
**W:M**	42 ± 0.58	37 ± 0.00	-	-	-	>500
**Leaves**						
**NH**	94 ± 0.58	70 ± 0.00	78 ± 1.53	49 ± 1.53	36 ± 3.61	67
**CH**	96 ± 1.00	90 ± 0.00	82 ± 2.52	78 ± 2.08	49 ± 1.53	35
**EA**	99 ± 0.58	94 ± 0.58	86 ± 1.00	48 ± 1.53	39 ± 1.15	71
**AC**	91 ± 1.00	80 ± 0.00	30 ± 0.58	21 ± 1.00	11 ± 1.00	190
**M**	69 ± 1.15	64 ± 1.00	58 ± 1.53	42 ± 1.53	30 ± 1.00	97
**ET**	75 ± 1.53	60 ± 1.53	50 ± 1.00	31 ± 1.00	19 ± 1.53	125
**W**	43 ± 1.53	20 ± 0.58	-	-	-	>500
**NH:EA**	94 ± 1.00	89 ± 1.00	89 ± 1.53	63 ± 2.52	51 ± 1.15	30
**NH:ET**	83 ± 1.53	76 ± 1.53	71 ± 1.53	67 ± 0.58	50 ± 0.58	32
**CH:M**	81 ± 1.53	63 ± 1.53	63 ± 2.00	51 ± 1.53	41 ± 1.53	62
**EA:M**	84 ± 1.00	78 ± 1.53	62 ± 2.00	39 ± 1.53	32 ± 2.00	76
**AC:M**	82 ± 1.53	73 ± 1.00	69 ± 1.53	63 ± 1.53	54 ± 2.65	30
**W:AC**	90 ± 0.00	74 ± 1.53	60 ± 1.00	38 ± 1.53	30 ± 0.58	83
**W:M**	93 ± 2.00	90 ± 0.58	58 ± 2.00	56 ± 1.53	28 ± 2.00	52

Negative control: DMSO. LC50 of Doxorubicin (positive control employed in the brine shrimp lethality assay) was 5.93 μg/mL. Values (mean ± SD) are the average of three samples of each plant extract, analyzed individually in triplicate (n = 1 × 3).

## Data Availability

Data sharing not applicable.

## Sample Availability

The crude extracts prepared for the current study are available from the authors.
